# Is semen analysis without strict criteria misleading decisions in IVF? A prospective systematic study

**Published:** 2018-07

**Authors:** Faduola Paul, Gbolahan Oladele Obajimi, Charles Olubukunmi Kolade

**Affiliations:** 1 *Vine Branch Fertility Clinic, Mokola, Ibadan, Nigeria.*; 2 *University of Ibadan, College of Medicine, Department of Obstetrics and Gynaecology and University College Hospital, Ibadan, Nigeria.*

**Keywords:** Semen analysis, Sperm morphology, Strict criteria, Fertilization, IVF, ICSI

## Abstract

**Background:**

Sperm morphology has been strongly linked to fertilization. This makes it an important component in semen analysis. They are usually assessed by world health organization (WHO) standard or Kruger strict criteria in in-vitro fertilization (IVF) centers all over the world. Sperm count, motility, and morphology together form the basis by which patients are allocated into IVF or intra-cytoplasmic sperm injection.

**Objective:**

This study aimed to compare fertilization rates in standard IVF from patients with normal sperm count and motility with and without morphological assessment by WHO guideline.

**Materials and Methods:**

In this prospective cohort study, sperm count, motility, and morphology of 504 men candidate for IVF program over a three years period in our center were evaluated in two groups: Group A (case group) included men with normal sperm count and motility but with a poor morphology and group B (control group) included men with normal sperm count, motility and morphology evaluated by WHO criteria. Fertilization rate in both groups were then analyzed after 16-18 hr post insemination.

**Results:**

Fertilization rate was higher in group B (p=0.028). Participants in group B, apart from having a normal sperm count (32.9±7.2) and motility (62.4±8.9), have a strict morphology of ≥30%. Our result has shown that spermatozoa in group B had a higher fertilization rate (71.4%). Though the sperm count (36.4±6.7) and motility (66.3±7.4) in group A were slightly higher (p=0.058 and p=0.060 respectively) than group B, the fertilization rate was lower.

**Conclusion:**

Our study showed that sperm morphology could be a very important consideration before decisions towards allocation of patients into IVF or intra-cytoplasmic sperm injection.

## Introduction

It has become a common practice worldwide that male infertility workup should start with a conventional semen analysis. This analysis involves assessing the semen sample for sperm count, motility, and morphology as a marker for male fertility potential (1). Despite the use of this test to accurately predict male with azoospermia, necrospermia, and teratozoospermia, it has failed considerably in indicating male fertility accurately in-vivo or in-vitro (2). To improve the power of predicting the accuracy of semen analysis, sperm count, motility and morphology were independently evaluated for best precision with little or no consensus (3-10). 

Regardless of the divides in opinion, semen analysis is generally agreed to be necessary in deciding whether in vitro fertilization (IVF) or intra-cytoplasmic sperm injection (ICSI) should be done (11-16). A sperm with good quality may fertilize with IVF or ICSI but a sperm with poor quality may fertilize with ICSI but not with IVF. In standard IVF insemination with <30% normal sperm morphology, fertilization rate averages between 0-30% (17). Men in this category should have their oocytes fertilized by ICSI (2, 18). 

This may not be possible in some IVF centers where semen quality is mainly assessed by sperm count and motility while morphology is roughly assumed under ×20 microscope. In poor resource country like Nigeria, the level of sophistication of IVF equipment and capabilities of clinical embryologist varies from clinic to clinic. It is therefore necessary to determine the morphology of sperm before deciding treatment options. 

This study seeks to compare fertilization rates in standard IVF done in participants with normal sperm count and motility with and without morphological assessment by WHO guideline. 

## Materials and methods

**Subject**

526 men referred to Vine Branch Fertility Center for IVF program between 2015 to 2017 were recruited in this prospective systematic study. 22 men were excluded because of lower count and/or motility on sperm sample pre and post preparation. The remaining sperm samples (n=504) were then processed by discontinuous density gradient using Allgrad wash (Lifeglobal, Connecticut, USA), Allgrad 90% and 45% (Lifeglobal, Connecticut, USA) and divided into two groups. Group A (case group) included 311 men with normal sperm count and motility but with a poor morphology (<30%) and group B (Control group) included 193 men with normal sperm count, motility, and morphology (≥30%) evaluated by WHO criteria. Also, the participant's partners were examined for hormonal level and baseline ultrasound examination. All couples with endometriosis, infertility due to immunological factors, and women’s age >37 yr old were excluded.

**Semen analysis**

Semen Samples were allowed to liquefy before processing and analysis were done according to the recommendations of the WHO (10). The volume of the semen was determined in a graduated universal tube (14 mL conical tube, Repromed, The Netherlands) and sperm concentration was determined using improved Neubauer hemocytometer (American Optical Company, Buffalo, NY) and expressed in millions/mL. The motility of the Sperm was assessed in at least 100 sperm and expressed as percent of motile sperm. Sperm morphology was assessed after Papanicolaou staining.

**Papanicolaou Staining**

Smears for morphology evaluation were prepared from the remaining samples used for sperm count and motility. They were then fixed and stained by Papanicolaou staining method for manual morphologic analysis (10). At least 200 spermatozoa were scored per slide with an oil immersion objective. The WHO criteria were used for scoring the spermatozoa. 30% cutoff value was used for normal morphologic findings while all borderline forms were considered abnormal. All slides were examined by one trained Medical Lab Scientist throughout the study.

**Sperm Preparation**

A sterile pipette was used to deliver 1mL of the “lower layer” (AllGrad 90 %, LifeGlobal, Connecticut, USA) into a 14mL conical tube (Repromed, The Netherlands). 1.0 mL of (AllGrad 45 %, LifeGlobal, Connecticut, USA) was placed on top of AllGrad 90% with another sterile pipette. 2.0 mL of the liquefied semen sample was carefully layered on the AllGrad 45% and spinned for 15 min in a centrifuge at 300×g. The density gradient layers were gently removed leaving behind the pellet. The pellet was then rinsed twice at 200xg for 5min after diluting with 2.0 mL of pre-warmed (AllGrad wash, LifeGlobal, Connecticut, USA). After the final centrifugation, the supernatant was removed and 2.0 mL of pre-warmed (AllGrad wash, LifeGlobal, Connecticut, USA) was carefully layered on the pellet and incubated in a CO_2 _incubator.

**Ovulation Induction**

Buserelin (Suprefact; Hoechst, Denmark) was administered for 10-15 days, and then a stimulation with daily injections of Menopur (Ferring, Parsippany, New Jersey (NJ), USA) or Gonal-F (Merck Serono, Geneva, Switzerland) was done followed by human chorionic gonadotrophin (Pregnyl; N.V. Organon, the Netherlands). Approximately 38 hr later, the oocytes were then aspirated using transvaginal ultrasound-guided retrieval. In the IVF cycles, each oocyte was inseminated with 200,000 sperm 4-5 hr after oocyte aspiration. All oocytes were then screened for fertilization 18-20 hr after insemination

**Ethical consideration**

Permission for this study was given by the local Institutional Review Board on 2 February 2015 (VBFC023). Informed consent was collected from each couple participating in the study for the use of their clinical data for research purposes.

**Statistical analysis**

Statistical evaluation was performed using student’s t-test on SPSS software (statistical package for the social science, version 16.0, SPSS Inc, Chicago, Illinois, USA). The p<0.05 were considered as statistically significant.

## Results

Sperm samples were analyzed from men in group A and B. The mean±SD of participant’s age was 39.6±0.7 yr old (40.3 yr in group A and 38.9 yr in group B). Demographic and sperm analysis variables are shown in table I. No statistical difference was found between group A and B in term of variables such as female and male age, sperm count, and motility as well as the number of oocytes and their maturity. Fertilization rate was significantly higher in group B than in group A (Table I). 41% of men in group A had fertilization rate ranging from 0 to 10% while only 3% of participants in group B had a fertilization rate of <50.

**Table I:** Parameters in group A and B

	**Group A**	**Group B**	**p-value**
Female age	33.6 ± 1.8	34.1± 1.5	0.052
Male age	40.3 ±3.4	38.9± 4.1	0.053
Number of oocytes	8.4± 2.3	7.2 ±1.9	0.052
Number of MII	7.3± 1.4	6.5± 5.0	0.054
Sperm concentration	36.4 ±2.7	32.9± 3.1	0.058
Motility	66.3± 5.2	62.4 ± 6.4	0.060
Morphology	17.9± 1.4	34.8 ±2.7	0.031
Fertilization rate	38.3± 0.7	71.4± 2.0	0.028

Data presented as mean

Student’s two-tailed t-test

MII: Metaphase II

## Discussion

Considering the cost of setting up an IVF center, new centers in Nigeria tend to start the practice of IVF without an ICSI machine. These centers do mainly conventional IVF until years later when they are able to get the funding to purchase an ICSI machine. Even after the purchase of an ICSI machine, it takes a while before their in-house embryologist becomes competent in the ICSI procedure. Most often, these centers seek the services of freelance embryologists to do their ICSI procedures whenever the need arise. Based on these and many other reasons, deciding whether a treatment will be done by IVF or ICSI is necessary when planning the treatment. It has already been recommended by many studies around the world that ICSI is the most effective treatment if sperm morphology <30% by WHO criteria (2, 18). Treating semen sample with <30% morphology by standard IVF may result in no or reduced fertilization. One important way of deciding treatment options is semen analysis. In most centers around Nigeria, semen analysis mainly involves sperm count, motility and morphology performed under x20 magnification. Under this magnification, sperm morphology is not accurately determined. Studies have linked poor sperm morphology to poor fertilization outcome (11). Having a sperm count, motility and morphology done according to the WHO guideline is likely to improve on fertilization.

This study is the first in Nigeria comparing sperm morphology by WHO guideline with standard IVF rate. The intention was to check if decisions made to recruit patients into IVF or ICSI with or without WHO guideline has no consequence on fertilization rate. Our emphasis was on fertilization because the new WHO manual tends to limit what we know as a normal sperm to spermatozoa that are able to fertilize an oocyte. 

In this study, we found that the fertilization was better in group B (p=0.028). Patients in this group have a normal sperm count, motility and morphology. Based on these normal parameters, this group served as control. Our result has shown that spermatozoa in this group had a higher fertilization rate (71.4%). This result is consistent with earlier studies on this topic (7, 8, 13-16). In group A, the sperm count and motility were better than those in group B. The fertilization and sperm morphology are however poor compared to group B. This result is not different from earlier studies were fertilization rate range from 0 to 30% with <30% normal sperm morphology by strict criteria (18). 

The table I showed no statistical differences in possible factors that could influence our outcome like female age, male age, number of oocytes aspirated, number of mature oocytes, sperm concentration and motility. We failed to statistically analyze some other factors such as hormonal level, baseline ultrasound, drugs used for ovarian induction, and the causes of infertility. This is due to the high degree of discrepancy within each factor that may hinder us from achieving the necessary numbers suitable for statistical analysis. To avoid influence from morphology result, smears used for the evaluation were prepared from the remaining samples used for sperm count, motility and the IVF procedure itself. We also ensure that all slides were stained and examined by one trained medical laboratory scientist throughout the study.

Our report has shown that assessing sperm morphology by strict criteria is very necessary when making decisions in IVF. This is important to avoid frustrating experience in which oocytes failed to fertilize 16-18 hr post insemination. In low income-country where patients fund their treatment independently, it is usually very difficult news for the embryologist to communicate. In conclusion, semen analysis without strict criteria is misleading decisions about how patients are allocated into IVF or ICSI.
